# Diagnosis and Treatment of Cystitis in Dogs: An Italian Survey

**DOI:** 10.3390/vetsci13050495

**Published:** 2026-05-20

**Authors:** Francesca Fidanzio, Isabella Tirelli, Simone Bertini, Alicia Maria Carrillo Heredero, Luigi Intorre, Ilaria Lippi, Veronica Marchetti, Cecilia Quintavalla, Andrea Corsini

**Affiliations:** 1 Department of Veterinary Sciences, University of Parma, 43126 Parma, Italy; isabella.tirelli@unipr.it (I.T.); simone.bertini@unipr.it (S.B.); aliciamaria.carrilloheredero@unipr.it (A.M.C.H.); cecilia.quintavalla@unipr.it (C.Q.); andrea.corsini@unipr.it (A.C.); 2 Department of Veterinary Medical Sciences, University of Pisa, 56122 Pisa, Italy; luigi.intorre@unipi.it (L.I.); ilaria.lippi@unipi.it (I.L.); veronica.marchetti@unipi.it (V.M.)

**Keywords:** urinary tract infections, subclinical bacteriuria, antibiotics, canine, antimicrobial stewardship, urine culture

## Abstract

Cystitis is a common condition in dogs and an important driver of antimicrobial prescription in small animal practice. Although international guidelines provide recommendations for diagnosis and management, their implementation in clinical settings remains variable. This survey-based study investigated the diagnostic and therapeutic approaches to canine cystitis among Italian veterinarians and explored differences between work settings. An anonymous online questionnaire was distributed nationwide between February and March 2024, yielding responses from 359 veterinarians. Of these, 63.2% worked in small clinics, 25.6% in large clinics, and 11.1% in veterinary hospitals. Diagnosis was most commonly based on the combination of symptoms, bloodwork, urinalysis, and abdominal ultrasound (45.1%) or on symptoms and urinalysis alone (43.7%). Urine culture and sensitivity testing (UCS) was performed in more than 50% of cases by 34.4% of small clinics, 55.4% of large clinics, and 72.5% of veterinary hospitals (*p* < 0.00001). Financial constraints, delayed results, and difficulty in urine collection were the main barriers. Overall, 56.2% of respondents included antibiotics as first-line therapy. Overall, antibiotic use did not differ among workplace categories, whereas fluoroquinolone prescription differed significantly (*p* = 0.021), being lower in veterinary hospitals than in small and large clinics. The most common treatment durations were 7 days (43.7%) and 10–14 days (43.2%). Management of subclinical bacteriuria was heterogeneous: probiotics (46.2%) and D-mannose (41.5%) were most frequently recommended, while 19.5% prescribed antibiotics and 19.8% opted for no therapy. These findings indicate variability in clinical practice, underuse of UCS, particularly in smaller clinics, and prescribing practices exceeding guideline recommendations.

## Simple Summary

Canine cystitis is a common reason for antimicrobial use in veterinary practice. This survey investigated how Italian veterinarians diagnose and manage cystitis and whether practices differ between clinics and hospitals. Responses from 359 veterinarians showed that 63.2% worked in small clinics, 25.6% in large clinics, and 11.1% in veterinary hospitals. Diagnosis was most often based on clinical signs combined with urinalysis, bloodwork, and abdominal ultrasound (45.1%), or on clinical signs and urinalysis alone (43.7%). Urine culture and sensitivity testing was performed in more than 50% of cases by 34.4% of small clinics, 55.4% of large clinics, and 72.5% of veterinary hospitals (*p* < 0.00001), with cost and delayed results reported as main barriers. Antibiotics were included in first-line therapy by 56.2% of respondents, with treatment durations being typically 7–14 days. Management of subclinical bacteriuria was variable, with some recommending probiotics or D-mannose, and others prescribing antibiotics or no treatment.

## 1. Introduction

Cystitis is a common diagnosis in dogs and significantly contributes to the antimicrobial prescription in veterinary medicine [1,2]. In dogs, bacterial urinary tract infections (UTIs) are the most frequent cause of cystitis, with *Escherichia coli* being the predominant pathogen [1]. Epidemiological studies indicate that up to 14% of dogs acquire a bacterial cystitis in their lifetime [3,4]. Female dogs are particularly predisposed to bacterial cystitis due to their shorter and wider urethra, which facilitates the ascent of bacteria from the perineal region [1,5].

The International Society for Companion Animal Infectious Diseases (ISCAID) provides practical guidelines for diagnosing and managing bacterial urinary tract infections in dogs and cats, distinguishing between sporadic and recurrent cystitis based on how often episodes occur [6]. Sporadic bacterial cystitis typically affects non-pregnant females or neutered males without known urinary tract abnormalities or relevant comorbidities, and is defined by fewer than three episodes in the previous 12 months [6]. In clinical practice, diagnosis is based on the presence of lower urinary tract signs, supported by urinalysis findings suggestive of a bacterial origin and confirmed by urine culture and susceptibility testing (UCS) [6,7].

The clinical symptoms reported by owners are collectively referred to as Lower Urinary Tract Signs (LUTS), which manifest through various observable behaviours and physiological changes reflecting underlying inflammation and discomfort [7]. The main complaints include stranguria, pollakiuria, dysuria, and hematuria [6].

The primary therapeutic option for canine cystitis in past decades has been antimicrobial therapy, which has contributed to the current problem of antimicrobial resistance [8,9]. The molecules prescribed by clinicians are various and contain both broad-spectrum antibiotics and critically important antimicrobials (CIA) [1,9]. Today, alongside antibiotics, clinicians often employ anti-inflammatory and nutraceutical supplements to reduce inflammation and promote a healthy urinary system [6,10].

Previous studies have shown variability in antimicrobial resistance patterns and prescribing practices in companion animal UTIs. A European multicenter study reported marked heterogeneity in resistance among uropathogens [11], while an Italian regional survey highlighted inconsistent prescribing behaviors and limited use of microbiological testing [12]. As a result, comprehensive nationwide data on the clinical management of canine cystitis in Italy are lacking. Addressing this gap is essential to evaluate current practices and identify potential discrepancies with guideline recommendations, particularly in the context of antimicrobial stewardship.

This survey aimed to describe how Italian veterinarians currently approach suspected canine cystitis in daily practice, with particular attention to diagnostic testing, antimicrobial selection, treatment duration, and management of subclinical bacteriuria. Additionally, we aimed to determine whether diagnostic approaches and antimicrobial treatment choices differ across various work environments.

We expected to observe a high rate of antimicrobial prescription for canine cystitis among veterinarians in Italy. Specifically, we hypothesized that the work setting may influence both the diagnostic approach to canine cystitis and the selection of antimicrobial treatments, and that in some cases the antimicrobial classes chosen and the duration of therapy would only partially align with current guideline recommendations.

## 2. Materials and Methods

### 2.1. Study Design

An observational, cross-sectional survey was performed to investigate current diagnostic and therapeutic approaches to canine cystitis among veterinarians in Italy. The questionnaire was created using Google Forms and distributed via professional forums and Provincial Veterinary Association mailing lists. Data collection was conducted between 12 February and 10 March 2024. The survey consisted of 11 mandatory, anonymous questions, including both multiple-choice and open-ended items (Supplementary Material S1).

The first section collected information on respondents’ professional settings (region, province, and type of facility). In accordance with national regulations, practices were classified as small clinics (SC; outpatient primary care), large clinics (LC; primary and secondary care for both inpatients and outpatients, without 24 h emergency service), or veterinary hospitals (VH; referral centers providing secondary and tertiary care with continuous emergency service).

Subsequent items addressed the diagnostic approach to canine cystitis, ranging from reliance on clinical signs alone to the use of complementary tests (e.g., bloodwork, urinalysis, abdominal ultrasound). Respondents were asked whether UCS was routinely performed and, if not, to report the main limiting factors.

The therapeutic section explored treatment choices, including antimicrobial agents, anti-inflammatory drugs, and adjunctive products (e.g., D-mannose, probiotics). Additional information was collected on the antimicrobial classes selected for empirical therapy and treatment duration. Participants were also asked whether UCS was routinely repeated after completion of antimicrobial treatment.

The final question investigated the management of subclinical bacteriuria, defined as a positive urine culture in the absence of clinical signs, with particular focus on treatment frequency and therapeutic strategies.

### 2.2. Statistical Analysis

Data were summarized descriptively and reported as counts and percentages. Differences among workplace categories were assessed using chi-square tests or Fisher’s exact tests, as appropriate, for contingency tables. When overall comparisons were significant or clinically relevant, pairwise comparisons between workplace categories were performed using two-sided Fisher’s exact tests, with Bonferroni correction for multiple comparisons. For within-workplace comparisons of short- versus long-term treatment duration criteria, two-sided binomial tests were used against an expected 50:50 distribution. Analyses were performed with GraphPad Prism 9 (GraphPad Software, San Diego, CA, USA), and statistical significance was set at *p* < 0.05.

## 3. Results

A total of 359 veterinarians completed the survey. The geographic distribution of respondents across the various Italian regions is reported in the (Supplementary Material S2).

Of these respondents, 227/359 (63.2%) worked in SC, 92/359 (25.6%) in LC, and 40/359 (11.1%) in VH.

Regarding the diagnosis of cystitis, 162/359 (45.1%) veterinarians reported using a combination of symptoms, bloodwork, urinalysis, and abdominal ultrasound, while 157/359 (43.7%) relied on symptoms and urinalysis alone. A smaller group (28/359, 7.8%) used symptoms, bloodwork, and urinalysis, and 12/359 (3.3%) made their diagnosis based solely on symptoms. No clinicians chose the combination of clinical signs and bloodwork.

**Figure 1:**
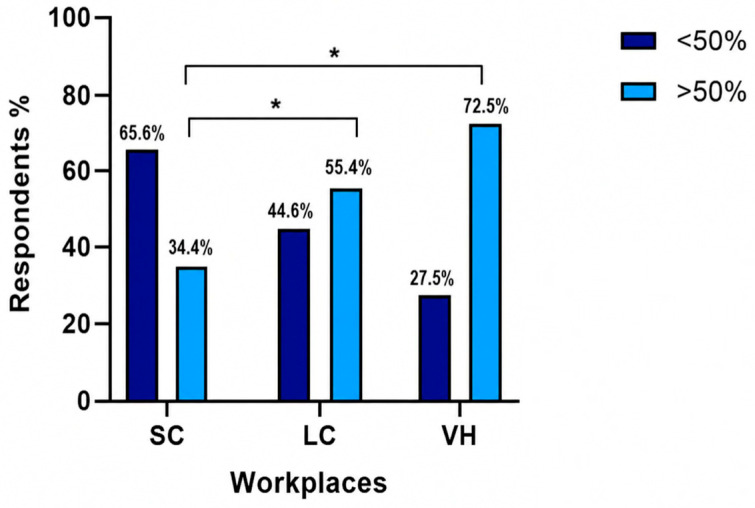
Percentage of respondents using UCS in more than 50% of patients across different work settings: SC, small clinics; LC, large clinics; VH, veterinary hospitals. * *p* < 0.05.

Urine culture and sensitivity testing was performed by 113/359 (31.5%) of respondents in less than 25% of cases, by 88/359 (24.5%) in 25–50% of cases, by 77/359 (21.4%) in 50–75% of cases, and by 81/359 (22.6%) in more than 75% of cases. The proportion of respondents who performed UCS in more than 50% of cases varied significantly across different work settings: 78/227 (34.4%) in SC, 51/92 (55.4%) in LC, and 29/40 (72.5%) in VH (overall *p* < 0.00001). Post hoc pairwise comparisons using two-sided Fisher’s exact tests with Bonferroni correction showed that this proportion was significantly higher in VH than in SC (*p* = 0.000024), but not significantly different between VH and LC (*p* = 0.245). The difference between LC and SC was also significant (*p* = 0.00196) (Figure 1).

The reasons cited for not performing UCS were ‘financial constraints of the owners’ (n = 268/359 [74.7%]), ‘delayed results’ (n = 73/359 [20.3%]), ‘difficulty in urine collection’ (n = 72/359 [20.1%]), ‘not necessary’ (n = 39/359 [10.9%]) and ‘challenges with shipping to an external laboratory’ (n = 16/359 [4.5%]).

Treatments prescribed by respondents are reported in Table 1. Overall, 202 of 359 respondents (56.2%) included antibiotics in their first-line treatment protocols, and there was no significant difference between workplaces in the use of antibiotics (*p* = 0.86). The empirically prescribed antibiotic classes are described in Table 2.

**Figure 2:**
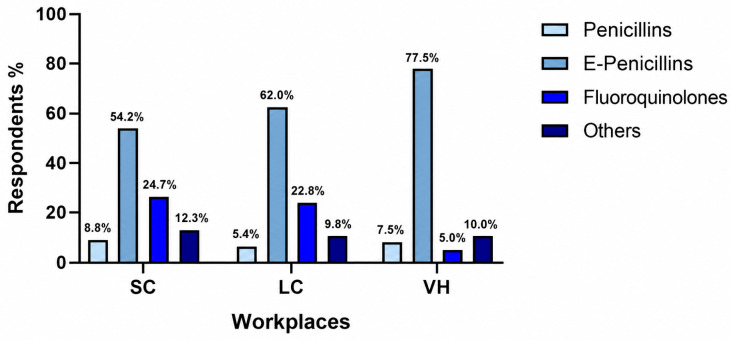
Prescription patterns for enhanced penicillins, fluoroquinolones, and penicillins across different workplaces. SC, small clinics; LC, large clinics; VH, veterinary hospitals.

**Figure 3:**
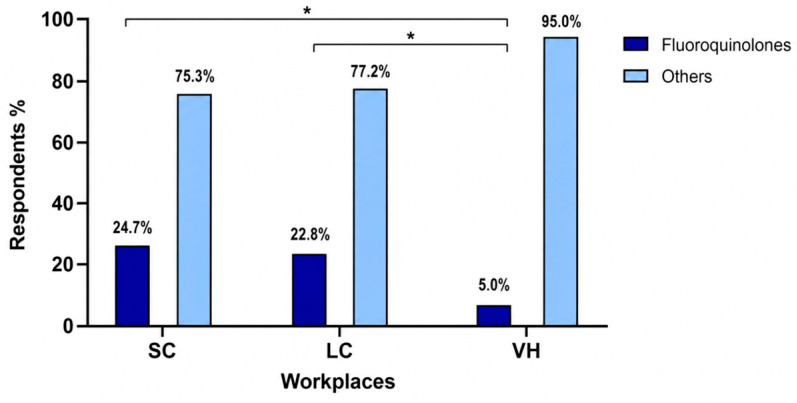
Fluoroquinolones prescription patterns across different workplaces. SC, small clinics; LC, large clinics; VH, veterinary hospitals. * *p* < 0.05.

The overall distribution of antimicrobial classes did not differ significantly among workplace categories (*p* = 0.095) (Figure 2). Enhanced penicillins were prescribed by 123/227 respondents in SC (54.2%), 57/92 in LC (62.0%), and 31/40 in VH (77.5%); fluoroquinolones by 56/227 in SC (24.7%), 21/92 in LC (22.8%), and 2/40 in VH (5.0%); and penicillins by 20/227 in SC (8.8%), 5/92 in LC (5.4%), and 3/40 in VH (7.5%). When fluoroquinolone prescription was analyzed separately, a significant difference was observed among workplace categories (*p* = 0.021) (Figure 3). Fluoroquinolones were prescribed less frequently in VH than in SC (*p* = 0.003) and LC (*p* = 0.012), whereas no significant difference was observed between SC and LC (*p* = 0.774).

**Figure 4:**
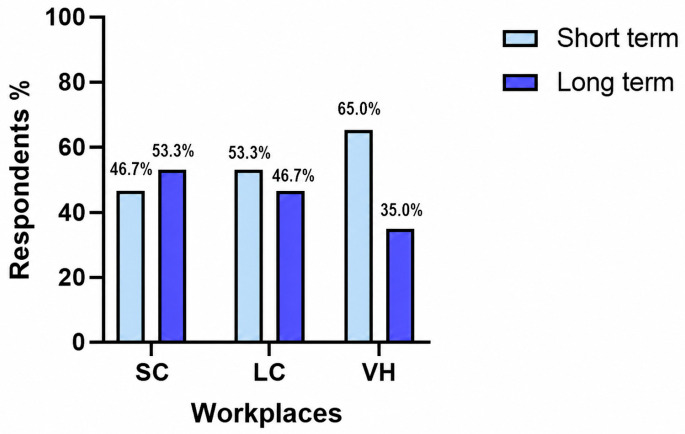
Distribution of short-term (≤7 days) and long-term (≥10 days) antibiotic treatment duration across different workplaces. SC, small clinics; LC, large clinics; VH, veterinary hospitals.

Regarding the duration of antibiotic therapy, 157/359 respondents (43.7%) prescribed antibiotics for 7 days, 155/359 (43.2%) for 10–14 days, 24/359 (6.7%) for 5 days or less, and 23/359 (6.4%) for more than 14 days. When treatment duration was categorized as short-term (≤7 days) or long-term (≥10 days), no significant association was observed between workplace category and treatment duration (*p* = 0.084) (Figure 4). Short-term duration was selected by 106/227 respondents in SC (46.7%), 49/92 in LC (53.3%), and 26/40 in VH (65.0%), whereas long-term duration was selected by 121/227 respondents in SC (53.3%), 43/92 in LC (46.7%), and 14/40 in VH (35.0%). Within each workplace category, no statistically significant difference was observed between short- and long-term duration: SC, *p* = 0.353; LC, *p* = 0.602; VH, *p* = 0.081.

Respondents were also asked about the management of subclinical bacteriuria; among the respondents, 166/359 (46.2%) prescribed probiotics, 149/359 (41.5%) recommended D-mannose, 71/359 (19.8%) opted for no therapy, 70/359 (19.5%) prescribed antibiotics, and 37/359 (10.3%) used anti-inflammatories. No statistically significant difference was observed between workplaces (*p* = 0.08).

## 4. Discussion

This Italian survey provides insights into canine cystitis from the perspective of clinicians, emphasizing the clinical presentation, diagnostic approaches, and treatment strategies for this condition. Overall, 56.2% of respondents reported the inclusion of antibiotics in their first-line treatment protocol for canine cystitis, with no significant differences between workplace settings. While empirical antimicrobial therapy is common in practice, international guidelines recommend that antimicrobial use and selection should be guided by clinical evidence and, where possible, supported by diagnostic testing such as UCS to promote antimicrobial stewardship and minimize the development of resistance; this approach is reflected in ISCAID recommendations for the diagnosis and management of UTIs in dogs and cats [6].

Although the overall antibiotic prescription rate was similar across SC, LC, and VH, the selection of specific antibiotic classes varied notably. Fluoroquinolones were prescribed more frequently in SC and LC compared to VH, whereas first-line drugs such as penicillins and enhanced penicillins were numerically more frequently reported in VH. This suggests that clinical environment, caseload complexity, and access to diagnostic resources (e.g., UCS) may influence antibiotic selection, with smaller clinics potentially relying more on broad-spectrum or higher-tier antimicrobials due to limited diagnostic capacity. These findings are consistent with previous survey-based and retrospective studies reporting that empirical fluoroquinolone use may vary according to practice type and clinical setting, with diagnostic support generally being more readily available in referral hospitals [4,13]. This highlights how the clinical environment and availability of diagnostic resources can shape antibiotic choice in canine urinary tract infections [4,13].

In the context of sporadic bacterial cystitis, ISCAID recommendations support the use of narrower-spectrum first-line antimicrobials, such as amoxicillin or trimethoprim-sulphonamide, when empirical treatment is considered appropriate. By contrast, fluoroquinolones and other higher-priority antimicrobials should generally be reserved for situations in which culture and susceptibility results indicate that first-line options are unsuitable, or when infection is recurrent or complicated [6]. Fluoroquinolones were prescribed more frequently in SC and LC than in VH. This finding should not be interpreted solely as a matter of individual clinical training, but rather as the result of multiple contextual factors, including diagnostic accessibility, turnaround time, economic constraints, clinical workflow, and variable implementation of antimicrobial stewardship strategies. This contrasts with guideline-based recommendations and highlights a worrisome gap between current practice and antimicrobial stewardship principles.

The high empirical use of antibiotics highlights the need for improved antimicrobial stewardship, particularly regarding fluoroquinolones, which the European Medicines Agency (EMA) classifies as critically important and recommends using with caution [14]. Overall, these observations emphasize the importance of routine urine culture and sensitivity testing to guide antimicrobial therapy, minimize unnecessary use of higher-tier antibiotics, and align clinical practice with evidence-based recommendations.

For otherwise healthy dogs with signs consistent with sporadic bacterial cystitis, ISCAID guidelines allow initial empirical management without mandatory urine culture in every case. Culture and susceptibility testing become more relevant when clinical signs persist, relapse occurs, or the clinical scenario suggests recurrent or complicated infection. Performing UCS in these cases allows for targeted antimicrobial selection, supports antimicrobial stewardship, and helps reduce the unnecessary use of broad-spectrum or critically important antibiotics [6].

For recurrent or complicated cystitis, ISCAID recommends performing quantitative UCS to confirm bacterial infection and guide targeted antimicrobial treatment. This approach ensures appropriate drug selection, monitors treatment efficacy, and contributes to minimizing antimicrobial resistance [6]. In our survey, UCS was underutilized overall: only a minority of clinicians reported performing UCS in more than half of cystitis cases. The proportion of veterinarians performing UCS more than 50% of the time increased with the level of care—from SC (34.4%) to LC (55.4%) and VH (72.5%)—indicating that larger and referral facilities may more consistently follow recommended diagnostic practices. This pattern may also partly reflect differences in case load, as referral centers are more likely to manage complex, recurrent, or refractory cases that warrant a more extensive diagnostic work-up.

Reported barriers to performing UCS, such as financial constraints, delayed results, and difficulty of urine collection, reflect practical challenges in implementing evidence-based diagnostics and may contribute to reliance on empirical antibiotic therapy. Although empirical antimicrobial therapy may reduce the initial cost of diagnostic testing, this approach may carry hidden costs related to inappropriate antimicrobial use, recurrence, treatment failure, and antimicrobial resistance. Therefore, the upfront cost of UCS should be balanced against its role in supporting targeted therapy and antimicrobial stewardship. Financial constraints may reflect both true owner-related limitations and perceived barriers by clinicians, highlighting the importance of owner education and communication regarding the long-term value of guideline-based diagnostics.

These findings align with scientific literature, which shows that multicenter surveys and retrospective studies have documented that UCS is frequently underutilized in primary care, particularly in smaller clinics, with testing often limited by cost or logistical constraints [4,8]. Adherence to recommended diagnostic practices tends to be higher in referral or larger facilities, mirroring the trends observed in our survey [4,8].

To reduce antimicrobial resistance, it is important not only to choose the right drug, preferably guided by urine culture, but also to optimize the duration of therapy [15,16]. According to ISCAID guidelines for sporadic bacterial cystitis in otherwise healthy dogs, short courses of 3–5 days with first-line antibiotics are generally sufficient when clinical signs resolve [6]. Longer courses are not recommended, as they do not improve outcomes and may increase the risk of antimicrobial resistance [15]. In our survey, the duration of antibiotic therapy exceeded the recommended duration: 43.7% of clinicians prescribed 7-day courses, while 43.2% prescribed 10–14-day courses. This pattern aligns with the literature, which shows that although short, evidence-based courses are effective for sporadic urinary tract infections, extended treatments are still commonly prescribed [16]. Overly long antibiotic courses increase selective pressure and the risk of antimicrobial resistance, highlighting the importance of adhering to guideline-recommended durations [15]. According to ISCAID guidelines, subclinical bacteriuria—defined as the presence of bacteria in urine without clinical signs of lower urinary tract disease—should not be treated with antibiotics in otherwise healthy dogs and cats. Antibiotic therapy is only indicated in patients at increased risk of complications, such as those undergoing urinary tract surgery, immunocompromised animals, or patients with urolithiasis [6]. In our survey, the management of subclinical bacteriuria showed considerable heterogeneity. While 41.5% and 46.2% of respondents recommended D-mannose and probiotics, respectively, 19.5% prescribed antibiotics, 10.3% used anti-inflammatories, and 19.8% opted for no therapy. Notably, nearly one in five clinicians still prescribed antibiotics, despite guideline recommendations to avoid treatment in asymptomatic patients. No significant differences were observed between workplaces, suggesting that this overprescription is widespread across different clinical settings. Evidence shows that not treating dogs with subclinical bacteriuria with antibiotics does not worsen clinical outcomes. In a prospective study of healthy dogs with subclinical bacteriuria, none of the animals developed clinical signs of urinary tract infection during follow-up, indicating that antibiotic therapy is unnecessary and does not improve outcomes [17].

Observational studies and surveys have also highlighted that the use of nutraceuticals or anti-inflammatories is highly variable, reflecting the lack of evidence-based, standardized protocols for non-antibiotic management of subclinical bacteriuria [15,16].

This study has some limitations. Firstly, despite the survey’s anonymity, respondents may have been influenced to choose answers they perceived as ‘correct’, introducing bias and potentially skewing our assessment of actual clinical practices among Italian veterinarians. Second, the term “canine cystitis” was used in reference to the ISCAID criteria for sporadic cystitis, but was not explicitly defined in the questionnaire. In dogs, cystitis most commonly reflects bacterial lower urinary tract infection, although this was not specified to respondents. This lack of clarity may have led to misinterpretations, affecting the reliability of responses. Additionally, the questionnaire did not explicitly specify that urinalysis should include urine sediment examination, and this cannot be assumed to be routinely performed by all respondents, potentially contributing to variability in answers and interpretation. Although compatible clinical signs were included in all response options regarding the diagnosis of canine cystitis, this lack of specification may have limited our ability to determine whether respondents consistently considered sediment evidence of inflammation and bacteriuria, as recommended by ISCAID guidelines. Finally, workplace comparisons were constrained by unequal respondent pools across SC, LC, and VH, which may have affected the statistical power of between-group comparisons, particularly for outcomes with *p*-values close to the significance threshold. Therefore, statistical differences and near-significant findings should be interpreted with caution.

## 5. Conclusions

This survey provides a comprehensive overview of the current diagnostic and therapeutic approaches to canine cystitis among Italian veterinarians. Empirical antibiotic use remains common, including in cases of subclinical bacteriuria, and the choice of antibiotic class and duration often exceeds guideline recommendations. Urine culture and sensitivity testing are underutilized, particularly in smaller clinics, and practical barriers, such as cost, logistics, and access to laboratory services, contribute to reliance on empirical therapy. The management of subclinical bacteriuria is highly heterogeneous, with some clinicians still prescribing antibiotics despite clear guideline recommendations.

These findings highlight the need for targeted educational interventions, improved access to diagnostic testing, and greater adherence to ISCAID guidelines. Educational efforts should specifically address the distinction between subclinical bacteriuria and bacterial cystitis, avoidance of antimicrobial therapy in asymptomatic cases, and appropriate use of first-line narrow-spectrum antibiotics and short treatment courses for sporadic cystitis.

## Figures and Tables

**Table 1:** Treatment choices reported by respondents, including antibiotics, anti-inflammatory drugs, and supplements.

Treatments	Answers
Antibiotic, anti-inflammatory, supplements	142/359 (39.6%)
Anti-inflammatory, supplements	137/359 (38.2%)
Antibiotic, anti-inflammatory	28/359 (7.8%)
Antibiotic, supplements	22/359 (6.1%)
Anti-inflammatory	10/359 (2.8%)
Supplements ^1^	10/359 (2.8%)
Antibiotic	10/359 (2.8%)

^1^ Dietary supplements were defined as nutritional or nutraceutical formulations intended to complement the diet by providing specific nutrients or bioactive substances that may support urinary tract health and function. Examples included in the questionnaire were D-mannose and probiotics.

**Table 2:** Empirically prescribed antibiotic classes.

Antibiotic Classes	Answers
Enhanced penicillins	211/359 (58.8%)
Fluoroquinolones	79/359 (22%)
Penicillins	28/359 (7.8%)
First-generation cephalosporins	11/359 (3%)
Third-generation cephalosporins	1/359 (0.3%)
No empirical prescription	29/359 (8.1%)

## Data Availability

The original contributions presented in this study are included in the article/Supplementary Materials. Further inquiries can be directed to the corresponding author.

## Supplementary Materials

The following supporting information can be downloaded at https://www.mdpi.com/article/10.3390/vetsci13050495/s1: File S1: translated questionnaire; File S2: answers between different regions.
