# Supplementary material for: Diagnosis and Treatment of Cystitis in Dogs: An Italian Survey
Source: Vet Sci. 2026 May 20;13(5):495. doi: 10.3390/vetsci13050495 (PMC13211681; doi:10.3390/vetsci13050495)
Supplement: Supplementary file 1 [file vetsci-13-00495-s001.zip › Supplementary material S1 (1).pdf]

Supplementary material S1: Translated questionnaire

1. What region do you work in?

    - Click the drop-down menu with regions

2. What province do you work in?

    - Click the drop-down menu with provinces

3. What facility do you work in?

    - Small veterinary clinic
    - Large veterinary clinic
    - Veterinary hospital

4. How do you diagnose cystitis in dogs?

    - Clinical signs
    - Clinical signs + bloodwork
    - Clinical signs + urinalysis
    - Clinical signs + bloodwork + urinalysis
    - Clinical signs + bloodwork + urinalysis + abdominal ultrasound

5. Do you perform urine culture and sensitivity tests?

    - In more than 75% of cases
    - In 50-75% of cases
    - In 25-50% of cases
    - In less than 25% of cases

6. What are the most common reasons why you do not perform urine culture and sensitivity tests? Choose a maximum of two options

    - Financial problems of the owner
    - Difficulty collecting urine
    - Difficulty sending sample to external laboratories
    - Late result
    - Do not consider it necessary

7. How do you typically treat cystitis in dogs?

- Antibiotic
- Anti-inflammatory
- Antibiotic + anti-inflammatory
- Complementary therapies (D-mannose/probiotics)
- Antibiotic + Complementary therapies (D-mannose/probiotics)
- Anti-inflammatory + Complementary Therapies (D-mannose/probiotics)
- Antibiotic + Anti-inflammatory + Complementary Therapies (D-mannose/probiotics)

8. Which antibiotic drug do you prescribe on an empirical basis (pending or in the absence of urine culture and sensitivity tests)?

    - Free answer

9. What is the average duration of antibiotic therapy you prescribe?

    - Less than or equal to 5 days
    - 7 days
    - 10-14 days
    - 14 days

10. Do you perform urine culture and sensitivity tests at the end of antibiotic therapy?

    - In more than 75% of cases
    - In 50-75% of cases
    - In 25-50% of cases
    - In less than 25% of cases

11. How do you treat subclinical bacteriuria*? You can choose more than one option

    - Antibiotic
    - Anti-inflammatory
    - D-mannose
    - Probiotics
    - No therapy

*Subclinical bacteriuria is defined as positive urine culture in patients who show no symptoms referable to lower urinary tract disease (e.g., stranguria, hematuria, pollakiuria).
