# Supplementary material for: Diagnosis and Treatment of Cystitis in Dogs: An Italian Survey
Source: Vet Sci. 2026 May 20;13(5):495. doi: 10.3390/vetsci13050495 (PMC13211681; doi:10.3390/vetsci13050495)

Supplementary material S2 – answers between different regions

Region	Answers
Emilia-Romagna	68
Lombardia	61
Veneto	52
Toscana	34
Piemonte	20
Lazio	19
Umbria	16
Campania	16
Abruzzo	14
Marche	14
Friuli-Venezia Giulia	10
Calabria	7
Trentino-Alto Adige	7
Liguria	7
Sicilia	6
Puglia	4
Basilicata	3
Molise	1
Sardegna	0
Valle d'Aosta	0

Total answers: 359

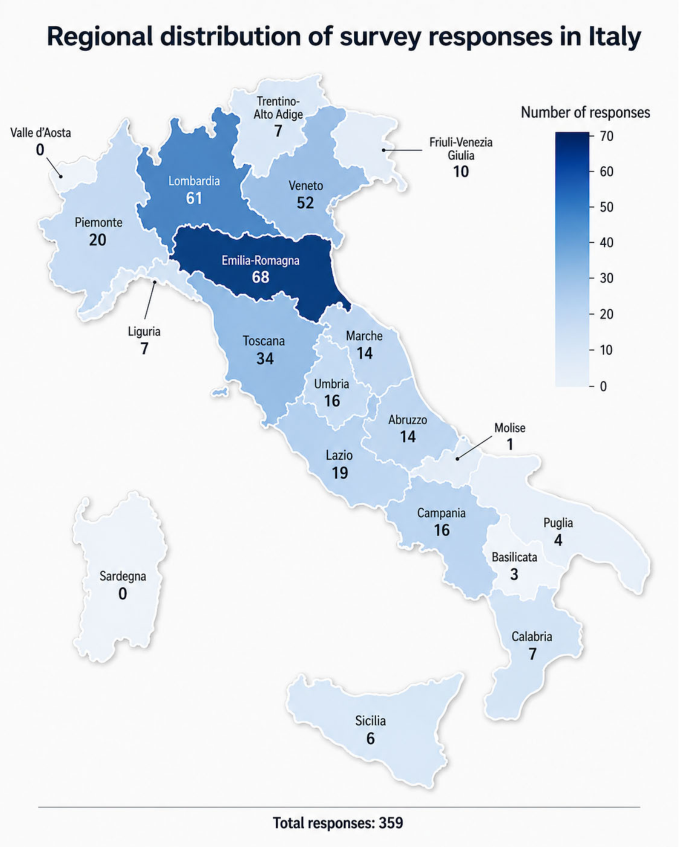

Supplement: Supplementary file 1 [file vetsci-13-00495-s001.zip › Supplementary material S2_R1.pdf]
